# FOXA1 prevents nutrients deprivation induced autophagic cell death through inducing loss of imprinting of *IGF2* in lung adenocarcinoma

**DOI:** 10.1038/s41419-022-05150-8

**Published:** 2022-08-16

**Authors:** Junjun Li, Yongchang Zhang, Li Wang, Min Li, Jianbo Yang, Pan Chen, Jie Zhu, Xiayu Li, Zhaoyang Zeng, Guiyuan Li, Wei Xiong, James B. McCarthy, Bo Xiang, Mei Yi

**Affiliations:** 1 Hunan Cancer Hospital and the Affiliated Cancer Hospital of Xiangya School of Medicine, Central South University, Changsha, 410013 Hunan China; 2 The Key Laboratory of Carcinogenesis of the Chinese Ministry of Health, Cancer Research Institute and School of Basic Medical Sciences, Central South University, Changsha, 410008 Hunan China; 3 The Key Laboratory of Carcinogenesis and Cancer Invasion of the Chinese Ministry of Education, Cancer Research Institute and School of Basic Medical Sciences, Central South University, Changsha, 410078 Hunan China; 4 Hunan Key Laboratory of Nonresolving Inflammation and Cancer, The Third Xiangya Hospital, Central South University, Changsha, 410013 Hunan China; 5 Department of Thoracic Surgery, Second Xiangya Hospital, Central South University, Changsha, 410011 Hunan China; 6 Department of Respiratory Medicine, Xiangya Lung Cancer Center; National Clinical Research Center for Geriatric Disorders, Xiangya Hospital, Central South University, Changsha, 410008 Hunan China; 7 Department of Laboratory Medicine and Pathology, Masonic Cancer Center, University of Minnesota, Minneapolis, MN 55455 USA

**Keywords:** Non-small-cell lung cancer, Targeted therapies

## Abstract

Lung cancer remains one of the most common malignancies and the leading cause of cancer-related death worldwide. Forkhead box protein A1 (FOXA1) is a pioneer factor amplified in lung adenocarcinoma (LUAD). However, its role in LUAD remains elusive. In this study, we found that expression of FOXA1 enhanced LUAD cell survival in nutrients deprived conditions through inhibiting autophagic cell death (ACD). FOXA1 bound to the imprinting control region of insulin-like growth factor 2 (*IGF2*) and interacted with DNA methyltransferase 1 (DNMT1), leading to initiation of DNMT1-mediated loss of imprinting (LOI) of *IGF2* and autocrine of IGF2. Blockage of IGF2 and its downstream insulin-like growth factor 1 receptor (IGF1R) abolished the protective effect of FOXA1 on LUAD cells in nutrients deprived conditions. Furthermore, FOXA1 suppressed the expression of the lysosomal enzyme glucocerebrosidase 1 (GBA1), a positive mediator of ACD, through ubiquitination of GBA1 enhanced by IGF2. Notably, FOXA1 expression in A549 cells reduced the efficacy of the anti-angiogenic drug nintedanib to inhibit xenograft tumor growth, whereas a combination of nintedanib with IGF1R inhibitor linsitinib or mTORC1 inhibitor rapamycin enhanced tumor control. Clinically, high expression level of FOXA1 protein was associated with unfavorable prognosis in LUAD patients of advanced stage who received bevacizumab treatment. Our findings uncovered a previously unrecognized role of FOXA1 in mediating loss of imprinting of IGF2, which confer LUAD cells enhanced survival ability against nutrients deprivation through suppressing autophagic cell death.

## Introduction

Lung adenocarcinoma (LUAD) is the most common histological type of lung cancer and one of the most common causes of cancer-related mortality worldwide [1]. Currently, the clinical outcomes of patients whose tumors are driven by somatically activated oncogenes, such as mutant epidermal growth factor receptor (*EGFR*), translocated anaplastic lymphoma kinase (*ALK*), and c-ros oncogene 1 receptor tyrosine kinase (*ROS1*), have been substantially improved by employing targeted therapies [2–6]. In addition to somatically activated mutants, several wild-type proto-oncogenes found to be amplified in LUAD [7], including NK2 homeobox 1 and telomerase, are desirable targets in cancer; however, some of these targets are not yet druggable.

The insulin-like growth factor II (IGF2) is a multifunctional growth hormone and plays essential roles in regulating cell growth, survival, migration and differentiation [8]. Upregulation of IGF2 and activation of the insulin-like growth factor 1 receptor (IGF-1R) pathway not only promote lung tumorigenesis [9, 10], but also are implicated in acquisition of therapy resistance [11]. Loss of imprinting (LOI) of *IGF2* is the major cause attributes to increase in IGF2 expression in LUAD [10, 12, 13]. However, it is not clear how LOI of *IGF2* is regulated in LUAD.

Forkhead box protein A1 (FOXA1) is a pioneer factor that loosens chromatin and facilitates the subsequent binding of lineage-specific transcription factors [14]. FOXA1 is a driver for prostate cancer and breast cancer [15, 16], whereas plays a tumor suppressive role in nasopharyngeal carcinoma [17, 18]. FOXA1 has been shown to act as an important factor in maintaining airway epithelial barrier integrity through regulating the expression of genes involved in epithelial development and tissue morphogenesis [19]. The pilot studies revealed that *FOXA1* is amplified in LUAD [20, 21]. Simultaneous deletion of *FOXA1* and *FOXA2* severely attenuates *K-Ras-*driven LUAD initiation and causes squamous cell identity shift [22]. The focal amplification of *FOXA1* in a fraction of human LUAD cases has attracted attention to its oncogenic function [22, 23]. However, the biological and clinical significance of FOXA1 expression in LUAD require further exploration.

In this study, we found that expression of FOXA1 confers survival advantage to LUAD cells in nutrients deprived conditions through suppressing autophagic dependent cell death. FOXA1 binds to and recruits DNMT1 to the imprinting control region (ICR) of *IGF2*, initiating LOI of *IGF2* and IGF2 autocrine in LUAD cells. Autocrine IGF2 prevents autophagic cell death of LUAD cells induced by nutrients deprivation through facilitating proteasomal degradation of lysosomal β-glucocerebrosidase-1 (GBA1), a positive regulator of autophagic cell death. Consequently, forced expression of FOXA1 hampered the efficacy of anti-angiogenesis reagent against A549 xenograft tumor, which could be restored by combinatory treatment with IGF1R inhibitor. Clinically, the expression levels of FOXA1 protein in LUAD samples associated to worse prognosis in LUAD patients of advanced stage who received anti-angiogenesis therapy.

## Materials and methods

### Cell culture and reagents

The LUAD cell lines A549, Calu-3, and PC-9 were purchased from the National Infrastructure of Cell Line Resource (Shanghai, China) and maintained in RPMI-1640 medium (Life Technologies, Grand Island, NY, USA) containing 10% fetal bovine serum and 1% penicillin-streptomycin. LUAD cell lines were routinely cultured at 37 °C (pH 7.4) in a humidified atmosphere consisting of 5% CO_2_ and 95% air.

Exogenous expression of FOXA1 in A549 and Calu-3 cells was achieved by infecting the cells with a FOXA1-expressing lentivirus. Loss of FOXA1 expression in PC-9 cells was achieved using CRISPR/Cas9 and RNA interference. A sgRNA targeted to exon 1 of FOXA1 was designed and introduced into PC-9 cells within the CRISPR system. After selection with puromycin, two independent clones showed complete depletion of FOXA1 protein and were used for further studies. Two lentiviral shRNAs were employed to silence endogenous FOXA1 in PC-9 cells. siRNA transfection was performed using Lipofectamine RNAiMAX reagent (Life Technologies) according to the manufacturer’s protocol. The sequences of sgRNAs, shRNAs, and siRNAs used are listed in Supplementary Table S1.

The EGFR-TKI Erlotinib (cat. no. OSI-774), Gefitinib (cat. no. ZD1839), the IGF1R inhibitor linsitinib (cat. no. S1091), rapamycin (cat. no. S1039), Fer-1 (cat. no. S7243), MG132 (cat. no. S2619), and nintedanib ethanesulfonate salt (cat. no. S5234) were purchased from Selleckchem (Shanghai, China). 3-methyladenine (3-MA, cat. no. HY-19312), Chloroquine (CQ, cat. no. HY-17589), and Nec-1 (cat. no. HY-15760) were purchased from MedChemExpress (Monmouth Junction, NJ, USA). z-VAD (cat. no. C1202) was purchased from the Beyotime Institute of Biotechnology (Shanghai, China).

### Cell viability assay

Single tumor cell suspensions were seeded into 96-well plates at a density of 2 × 10^3^ cells/well in 200 μL medium with 10% FBS. Then cells were incubated at 37 °C (pH 7.4) in a humidified incubator and allowed to grow for indicated time. Cell viability was measured by Cell Counting Kit-8 (CCK-8) according to previous demonstration [24]. The sample size of each group is shown in the figure legend. The data are expressed as mean±standard deviation (SD). No data were excluded from the analysis.

### Colony formation assay

Colony formation assays were performed as previously described [25, 26]. Briefly, LUAD cells were plated in 6-well plates at a density of 1 × 10^3^ cells/well. After 24 h, the cells were cultured in fresh complete medium or PBS for 24 or 48 h and then further cultured in complete medium for 10–14 days until colony formation. After fixation with 4% paraformaldehyde, the colonies were visualized using 0.1% crystal violet and counted. Colony formation assays were performed in triplicate. The data are expressed as mean ± SD.

### RNA extraction and real-time reverse-transcription (RT) PCR

TRIzol reagent (Life Technologies) was used to extract total cellular RNA. The residual genomic DNA was digested with RNase-free DNase I (Takara, Beijing, China), and complementary DNA was then synthesized using a RevertAid First Strand cDNA Synthesis Kit (Thermo Fisher Scientific, Beijing, China). Quantitative RT-PCR assays were performed using SYBR Green reagent (Bimake, Shanghai, China) on a CFX96 Touch Real-Time PCR Detection System (Bio-Rad Laboratories, Richmond, CA, USA). The relative expression levels of genes were calculated according to the 2^−ΔΔCT^ method. The primers used this study are listed in Supplementary Table S2. RT-PCR assays were performed in triplicate. The data are expressed as mean ± SD.

### RNA-Seq

RNA samples used for RNA-seq were prepared as described earlier [17]. The mRNA expression profiles were determined using RNA-seq on an Illumina HiSeq platform (San Diego, CA, USA). The NOISeq method was employed to determine differentially expressed genes with a fold change greater than or equal to 2 [27]. The datasets used in this paper are freely available upon request.

### Methylation-specific PCR

Genomic DNA (gDNA) was extracted using an E.Z.N.A. Tissue DNA Kit (Omega Bio-tek, Changsha, China). Cellular gDNA was then modified with sodium bisulfite using an EpiArt DNA methylation Bisulfite kit (Vazyme Biotech Co. Ltd, Beijing, China) according to the manufacturer’s instructions. The methylation levels of ICR DNA at the H19/IGF2 locus were assessed using methylation-specific PCR as described previously [28].

### Western blot analysis

Cellular proteins were prepared using RIPA buffer as described previously [25]. One-dimensional sodium dodecyl sulfate polyacrylamide gel electrophoresis (SDS-PAGE) was employed to separate cellular proteins. After transferring to polyvinylidene fluoride membranes (Millipore, Billerica, MA, USA) and blocking with 5% nonfat milk, the membrane-bound proteins were visualized using horse radish peroxidase-conjugated secondary antibodies that recognized the primary antibodies. The chemiluminescent signal was developed using an ECL detection system (Thermo Fisher Scientific) and recorded using a ChampChemi500 system (Sagecreation, Beijing, China). The primary and secondary antibodies used in this study are listed in Supplementary Table S3. The full length uncropped original western blots used in their manuscript was provided as the original data file.

### Co-IP assays

Relevant protein-protein interactions were assessed using Co-IP assays. Cells were lysed with an immunoprecipitation buffer containing Tris (50 mM, pH 7.6), ethylenediaminetetraacetic acid (1 mM), NaCl (50 mM), 1% Triton X-100, NaF (10 mM), Na_3_VO_4_ (1 mM), and protease inhibitor cocktail (Roche). Proteins complexes were captured using specific antibodies at 4 °C overnight and precipitated using protein G-conjugated agarose. After washing with PBS four times, the protein complexes were separated by SDS-PAGE and detected with corresponding antibodies using western blot assays.

### ChIP assays

The binding of proteins to DNA and the status of histone modifications were assessed using ChIP assays. Briefly, chromatin was crosslinked using 1% formaldehyde and then fragmented by sonication. The sheared chromatin was subjected to immunoprecipitation with specific antibodies. The precipitated protein-DNA adducts were reversed, and the resulting DNA was purified for PCR analyses using phenol-chloroform extraction. Primers used for ChIP-PCR analyses are listed in Supplementary Table S2. Data for ChIP-seq of FOXA1, H3K4me1, and H3K27ac from MCF7 cells were retrieved from the ENCODE program (GSE105305 for FOXA1, GSE86714 for H3K4me1, and GSE96352 for H3K27ac).

### Immunofluorescence assays

Immunofluorescence assays were performed as described previously [25]. Briefly, the cells were washed with PBS and then fixed with 4% paraformaldehyde at 37 °C for 60 min. After permeabilizing with 0.1% Triton X-100 and blocking with 5% bovine calf serums at room temperature, the cells were stained with the indicated primary antibodies and Cy3 or fluorescein isothiocyanate-conjugated secondary antibodies. Nuclei were visualized using 4′,6-diamidino-2-phenylindole. A laser confocal fluorescence microscope (Olympus) was employed to record the fluorescence images in these experiments.

### Measurement of autophagic flux

Autophagic flux was measured using a fluorescent probe, the tandem fluorescent-tagged LC3, as described previously [29]. The stubRFP-sensGFP-LC3 (GeneChem Corporation, Shanghai, China) fluorescent probe was introduced into LUAD cells using a lentiviral system. In lentivirus-infected cells, yellow puncta (i.e., RFP^+^GFP^+^) represented autophagosomes, whereas red puncta (i.e., RFP^+^GFP^−^) represented autolysosomes. Increased yellow puncta indicated enhancement of autophagic flux.

### Tumor tissue samples and immunohistochemical staining

A cohort of 81 LUAD from patients of advanced stage was recruited between January 2001 and October 2004 from the Pathology Department of the Hunan Cancer Hospital (Hunan, PR China). Among these patients, 51 patients received treatment with EGFR-TKIs and bevacizumab, 30 patients received EGFR-TKIs treatment alone. FOXA1 protein levels were evaluated using immunohistochemical staining as previously described [17, 25]. The immunoreactive score was calculated as the sum of staining intensity (scores: negative = 0, weak = 1, moderate = 2, or strong = 3) and frequency of staining of tumor cells (scores: <10% = 1, 10–50% = 2, >50% = 3). Immunoreactive scores in the range 0–2 and 3–6 were recorded as low and high expression, respectively. The use of clinical samples was approved by the Institute Research Ethics Committee of Central South University and consent forms to participate were obtained from each patient.

### Xenograft tumor formation assays

A xenograft tumor model was employed to evaluate the effects of FOXA1 expression on A549 cell tumorigenicity and responses to antitumor treatments. Mice were randomized into different groups before tumor cell inoculation (10 in each group). Briefly, 1 × 10^6^ cells in 0.2 mL culture medium were subcutaneously injected into 5-week-old male BALB/c nude mice (Shanghai SLAC Laboratory Animal Co. Ltd., Shanghai, China). When the xenograft tumors were visible, the mice were randomly assigned to different groups and treated with the indicated antitumor agents. Tumor diameter was monitored using calipers, and tumor volume (mm^3^) was calculated according to a formula: 0.5 × (the shortest diameter)^2^ × (the longest diameter). At the endpoint of the experiments, the xenograft tumors were excised from the mice, weighed, and fixed in 4% saline-buffered formalin. The experiment was performed as investigator-blinded. All animal experiments were performed according to the Animal Ethics Committee of Central South University. Paraffin-embedded xenograft tumors were sectioned at 4-μm thickness and subjected to H&E or immunohistochemical staining. The sample size of each group is shown in the figure legend. No data were excluded from the analysis.

### Statistical analysis

All experiments were performed with no fewer than three biological replicates. The data are presented as mean ± SD. The quantitative variable differences were analyzed using Student’s *t* test. All results are presented as mean ± SD. Two-way analysis of variance was utilized to compare xenograft tumor growth between different groups. Kaplan–Meier plotter was used to analyze the associations between FOXA1 expression and progression-free survival in patients with LUAD who received treatment with EGFR-TKIs and bevacizumab. Statistical histograms were developed using Prism 5.0 (GraphPad Software, CA, USA) and SPSS v17.0 software (SPSS, Chicago, IL, USA). Results with *P* values < 0.05 were recognized as statistically significant.

## Results

### FOXA1 expression conferred a survival advantage to LUAD cells in vitro in nutrients deprived conditions

**Fig. 1:**
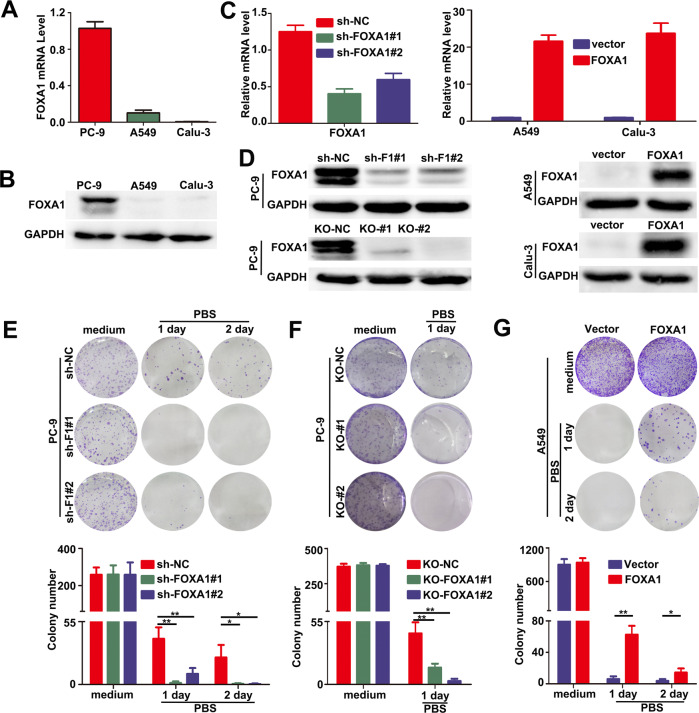
FOXA1 expression conferred a survival advantage in LUAD cells in starvation. **A** The endogenous mRNA levels of FOXA1 in LUAD cells were determined using RT-PCR. Mean ± SD, *n* = 3. **B** The endogenous protein levels of FOXA1 in LUAD cells were determined using western blotting. **C** The mRNA levels of FOXA1 in loss-of-function or gain-of-function LUAD cell models were measured using RT-PCR. Mean ± SD, *n* = 3. **D** The protein levels of FOXA1 in loss-of-function or gain-of-function LUAD cell models were measured using western blotting. **E**, **F**, **G** Cell survival measured using colony formation assays. Mean ± SD, *n* = 3. *P < 0.05; **P < 0.01; *P < 0.001.

By analyzing RNA-seq data from The Cancer Genome Atlas (TCGA) database, we found that the mRNA levels of *FOXA1* were significantly elevated in LUAD samples (Fig. S1), suggesting it might play an oncogenic role during LUAD development and progression. We measured FOXA1 expression levels in various LUAD cell lines. As shown in Fig. 1A, B, FOXA1 mRNA and protein levels were high in PC-9 cells, but weak or undetectable in A549, Calu-3 cells. We then employed clustered regularly interspaced short palindromic repeats (CRISPR)/Cas9 and targeted short hairpin RNAs (shRNAs) to establish FOXA1 loss-of-function models in PC-9 cells. Genome sequencing indicated successful gene editing at *FOXA1* locus by CRISPR/Cas9 technique (Fig. S2). Alternatively, a *FOXA1* cDNA-expressing lentivirus was used to generate gain-of-function models in A549 and Calu-3 cells. The mRNA and protein levels of FOXA1 in the gain- or loss-of-function models were determined using quantitative polymerase chain reaction (qPCR) or western blotting (Fig. 1C, D). To our surprise, gain- or loss-of-function of FOXA1 in LUAD cells did not significantly affect cell growth, migration, or invasiveness under normal culture conditions (Fig. S3A, B). Furthermore, expression of FOXA1 in LUAD cell lines also did not affect cells sensitivity to erlotinib and gefitinib (Fig. S3C), two EGFR-TKIs broadly used in LUAD patients. Unexpectedly, when A549 cells were mistakenly subjected in phosphate-buffered saline (PBS) for 24 h in an experiment, we observed that FOXA1-expressing A549 cells exhibited survival advantage than the control cells, prompting us to consider that FOXA1 might confer survival advantage to LUAD cells upon nutrient deprivation. To this end, we treated tumor cells by subjecting tumor cells in nutrient-free PBS for 24 or 48 h. Colony formation assays revealed that loss of FOXA1 expression using CRISPR/Cas9 or shRNA in PC-9 cells resulted in impaired cell survival in nutrient-free PBS (Fig. 1E, F). By contrast, forced expression of FOXA1 in A549 cells conferred a significant survival advantage in PBS (Fig. 1G). Nevertheless, forced expression of FOXA1 in Calu-3 cells showed no significant effect on cell survival both under nutrient-rich and nutrients deprived conditions (Fig. S4), which might be due to lack of downstream effectors of FOXA1 in this cell context.

### FOXA1 suppressed autophagic flux in LUAD cells upon nutrients deprivation

**Fig. 2:**
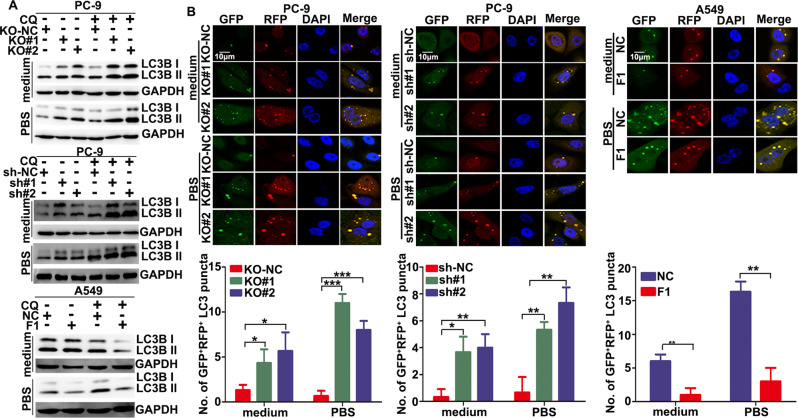
FOXA1 expression inhibited stress-induced autophagic flux in LUAD cells. **A** Autophagic flux in LUAD cells was evaluated using western blotting. CQ (10 μM). **B** Autophagic flux in LUAD cells was estimated using tandem fluorescent-tagged LC3 (stubRFP-sensGFP-LC3) reporter. The yellow puncta (RFP^+^GFP^+^) represent autophagosomes, and the red puncta (RFP^+^GFP^−^) represent autolysosomes. CQ (10 μM). Mean ± SD, *n* = 3. *P < 0.05; **P < 0.01. Scale bar: 10 μm.

Nutrients deprivation may induce autophagy, which can be either cytoprotective or cytotoxic. We assumed that FOXA1 might protect LUAD cells in nutrients deprived conditions through regulating autophagy induction in LUAD cells. As shown in Fig. 2A, CRIPSR/Cas9- or shRNA-dependent loss of FOXA1 expression in PC-9 cells slightly increased autophagic flux in complete culture medium. When cells were cultured in nutrient-free PBS, loss of FOXA1 in PC-9 cells led to a dramatic increase in autophagic flux, as evidenced by the increase in basal levels of LC3-II and a further increase in LC3-II upon chloroquine (CQ) treatment. By contrast, overexpression of FOXA1 in A549 cells resulted in a mild reduction of autophagic flux in complete culture medium. Upon CQ treatment, there was a significant increase in LC3-II protein levels in vector control A549 cells cultured in PBS, whereas this increase in LC3-II protein levels was not observed in FOXA1-expressing A549 cells. Autophagic flux was further monitored using a tandem fluorescent-tagged LC3 (stubRFP-sensGFP-LC3). As shown in Fig. 2B, there were a slight increase in yellow LC3 puncta (red fluorescent protein [RFP]^+^-green fluorescent protein [GFP]^+^-LC3) in FOXA1-deficient PC-9 cells when cultured in complete medium. When cells were subjected to PBS starvation, more yellow LC3 puncta appeared in PC-9 cells lacking FOXA1 expression. However, forced expression of FOXA1 in A549 cells slightly reduced the number of yellow LC3 puncta under nutrient-rich conditions. Prolonged nutrients deprivation significantly increased the number of yellow LC3 puncta in vector control A549 cells, but not in FOXA1-expressing A549 cells (Fig. 2B). These data indicate that nutrients deprivation-induced autophagic flux in LUAD cells, whereas FOXA1 expression in LUAD cells suppressed autophagic flux induced by nutrients deprivation.

### FOXA1 suppresses autophagic dependent cell death induced by nutrients deprivation in LUAD cells

**Fig. 3:**
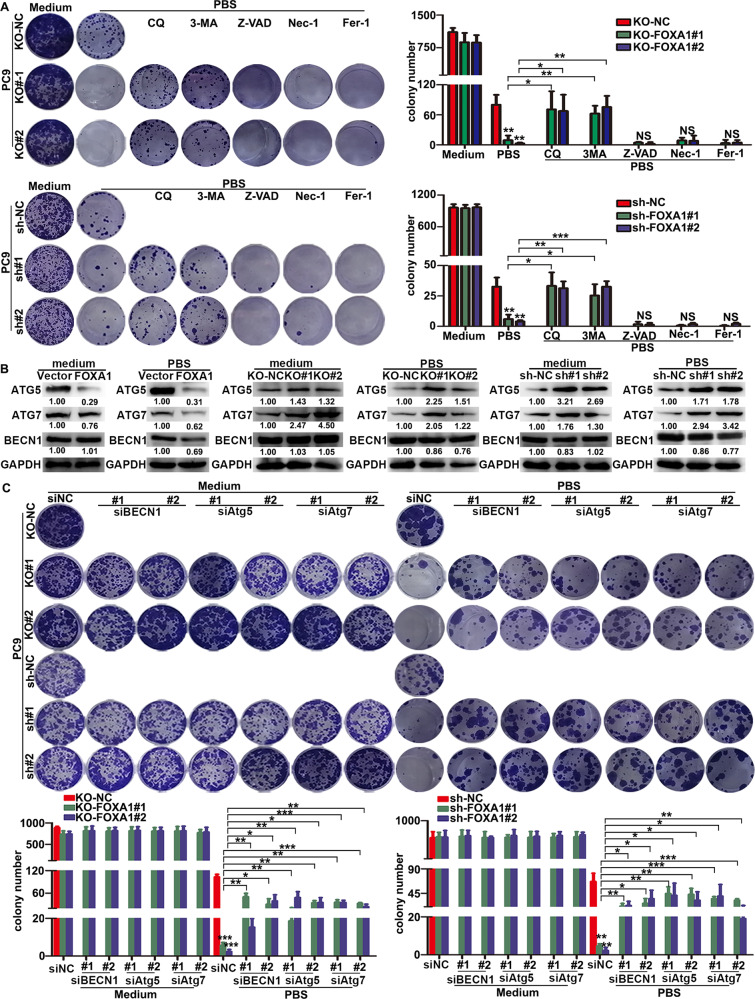
Loss of FOXA1 promoted autophagic cell death in LUAD cells under metabolic stress conditions. **A** Colony formation assays were used to evaluate cell survival in FOXA1-depleted or FOXA1-silenced PC-9 cells upon treatment with specific cell death inhibitors. CQ (10 μM), 3-MA (2 mM), Z-VAD (20 μM), Nec-1 (20 μM), Fer-1 (10 μM). Mean ± SD, *n* = 3. **B** The protein levels of autophagy-related genes were determined by western blot. **C** Colony formation assays were used to evaluate cell survival in FOXA1-depleted or FOXA1-silenced PC-9 cells upon inhibition of autophagy-related genes. Mean ± SD, *n* = 3. *P < 0.05; **P < 0.01; ***P < 0.001, NS not significant.

Autophagy may play a prosurvival or prodeath role in different contexts [30, 31]. In order to define whether increased autophagic flux play a prosurvival or prodeath role in FOXA1-deficient PC-9 cell in PBS, we treated the cells with 3-methyladenine (3-MA) or CQ, two chemical inhibitors of autophagy. As shown in Fig. 3A, supplementing cells with either 3-MA or CQ enhanced the survival of FOXA1-deficient PC-9 cells in PBS, whereas inhibitors of apoptosis (z-VAD), necroptosis (Nec-1), ferroptosis (Fer-1) failed to rescue survival of FOXA1-deficient PC-9 cells in nutrients deprived conditions, suggesting that nutrients deprivation may induce death in FOXA1-deficient PC-9 cells via an autophagy-dependent mechanism. Western blot assays indicated forced expression of FOXA1 in A549 cells reduced the protein levels of autophagy-related gene (ATG) 5 and ATG7 either in nutrients-rich or nutrients deprived conditions, whereas loss of FOXA1 in PC-9 cells exerted opposite effects (Fig. 3B). However, FOXA1 did not significantly affect the protein level of beclin 1 in LUAD cells (Fig. 3B). We then silenced beclin 1, ATG5, or ATG7 in FOXA1-deficient PC-9 cells (Fig. S5). As shown in Fig. 3C, inactivation of the autophagic machinery by silencing ATG5, or ATG7 in FOXA1-deficient PC-9 cells had minimal effect on cell survival under nutrients-rich conditions, but effectively rescued the survival of FOXA1-deficient PC-9 cells in nutrients deprived conditions. Though FOXA1 did not significantly affect the protein level of beclin 1, disruption of autophagic machinery by silencing beclin 1 also enhanced the survival of FOXA1-deficient PC-9 cells in nutrients deprived conditions, indicating that the intact autophagic machinery is required for nutrients deprivation-induced cell death of FOXA1-deficient LUAD cells. Thus, these results collectively indicated that FOXA1 expression in LUAD cells enhanced survivability in nutrients deprived conditions through inhibiting autophagic dependent cell death.

### FOXA1 directly induced loss of IGF2 imprinting in LUAD cells

**Fig. 4:**
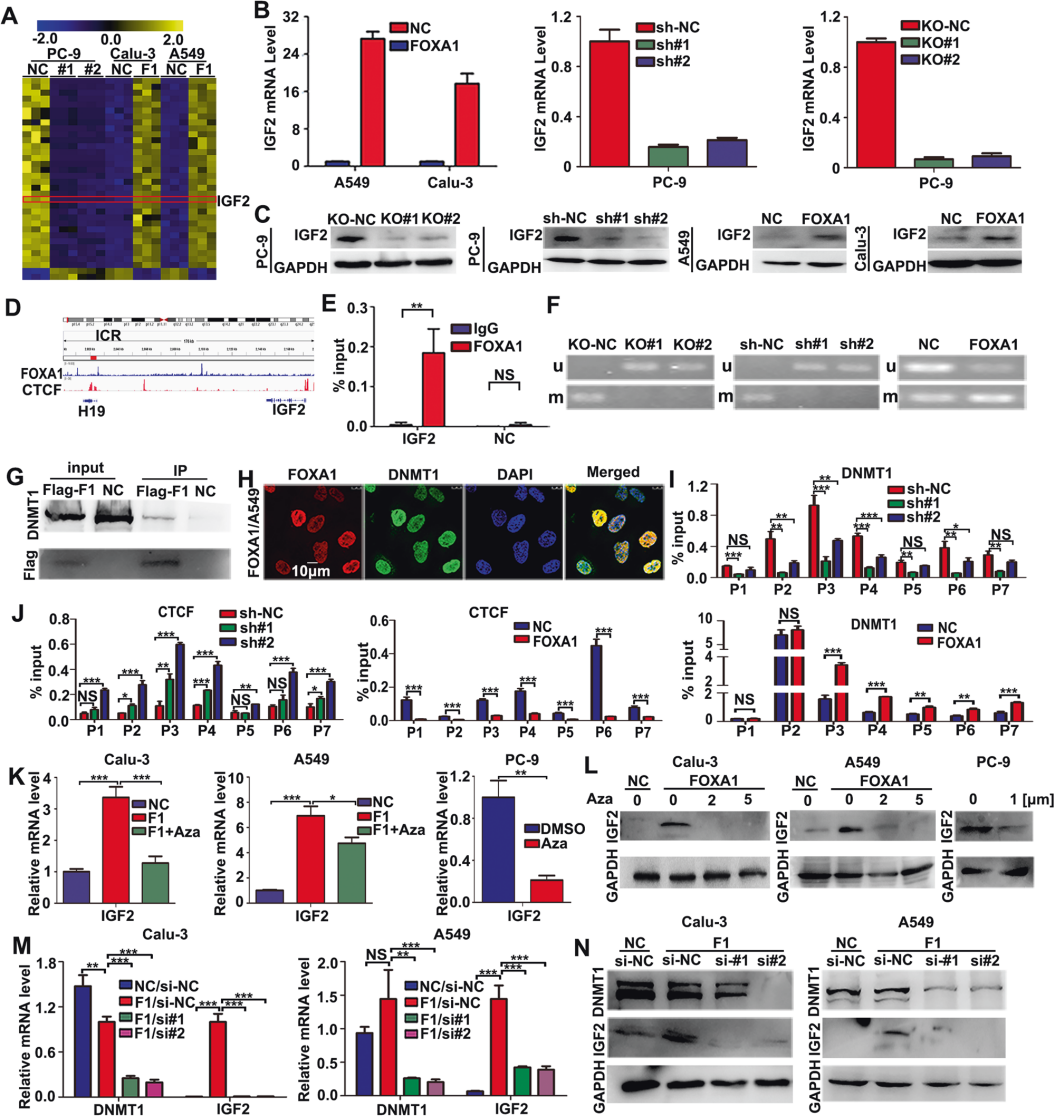
FOXA1 interacted with DNMT1 and mediated the loss of imprinting of *IGF2* in LUAD cells. **A** Heat map of differentially expressed genes induced by gain or loss of function of FOXA1 in LUAD cells. **B** RT-PCR assays. **C** Western blot assays. **D** ChIP-seq data visualized using IGV software. **E** FOXA1 binding to IGF2-ICR measured using ChIP-qPCR assays. Mean ± SD, *n* = 3. **F** Methylation status of IGF2-ICR LUAD cells as evaluated by MSP assays. **G** Co-immunoprecipitation assays. **H** Co-localization of FOXA1 with DNMT1 visualized using immunofluorescence assays. Scale bar: 10 μm. **I**, **J** DNMT1 or CTCF binding to the CpG sites of IGF2-ICR determined by ChIP-qPCR. Mean ± SD, *n* = 3. **K**, **L** Effects of 5-Aza on IGF2 mRNA and protein levels measured by qPCR and western blot assays. Mean ± SD, *n* = 3. **M**, **N** Effects of silencing DNMT1 on IGF2 mRNA and protein levels measured by qPCR and western blot assays. Mean ± SD, *n* = 3. *P < 0.05; **P < 0.01; ***P < 0.001, NS not significant.

To define the upstream signaling molecules in regulating autophagic cell death by FOXA1 in LUAD cells, we investigated the differentially expressed genes caused by gain- or loss-of-function of FOXA1. Totally, there were 34 genes consistently regulated by FOXA1 in different LUAD cell lines. Among the 34 target genes, 32 genes were positively regulated, whereas the other two genes were negatively regulated by FOXA1 in LUAD cells (Fig. 4A) (Table S4). IGF2, a prosurvival factor which is usually employed by cancer cells in response to different stressful stimuli [32, 33], was repressed by loss of FOXA1 but upregulated by gain of FOXA1. The qPCR and western blot assays confirmed that IGF2 mRNA and protein levels were positively regulated by FOXA1 in LUAD cells (Fig. 4B, C).

*IGF2* is an imprinting gene regulated by the methylation status of a cis-element known as the imprinting regulatory region (ICR). Biallelic expression of IGF2 is frequently observed in cancers due to DNA methyltransferase 1 (DNMT1)-mediated de novo methylation of the ICR, a process called loss of imprinting (LOI) [11, 34]. Chromatin immunoprecipitation sequencing (ChIP-seq) data suggested that there was a FOXA1 binding site close to the seven CCCTC-binding factor (CTCF) binding sites at the H19/ICR locus (Figs. 4D, S6). ChIP-qPCR assays confirmed the specific binding of FOXA1 to the ICR locus in A549 cells (Fig. 4E). We then examined whether FOXA1 binding affected the methylation status of the CTCF binding sites at ICR locus, which is critical to LOI of *IGF2* in lung cancer [11]. Methylation-specific PCR assays showed that the methylation level of IGF2-ICR locus decreased, whereas that of unmethylated IGF2-ICR increased upon loss of FOXA1 in PC-9 cells (Fig. 4F). By contrast, forced expression of FOXA1 in A549 cells led to reduced levels of unmethylated IGF2-ICR, but increased levels of IGF2-ICR methylation (Fig. 4F). Thus, our data indicated that FOXA1 directly bound to a site adjacent to the CTCF binding sites at IGF2-ICR locus, leading to LOI of *IGF2*.

Because LOI of *IGF2* is known to be mediated by DNMT1 [11, 34], we asked whether FOXA1 initiate LOI of *IGF2* in LUAD cells through interacting with DNMT1. As expected, co-immunoprecipitation (Co-IP) assays revealed that endogenous DNMT1 was immunoprecipitated with Flag-FOXA1 protein in A549 cells (Fig. 4G). Immunofluorescence assays indicated that FOXA1 co-localized with DNMT1 protein in A549 cells (Fig. 4H). We employed different primers sets which were used in a previous study [11] to evaluating the effects of FOXA1 on CTCF or DNMT1 binding to the seven CTCF binding sites at IGF2-ICR (Fig. S6). Loss of FOXA1 in PC-9 cells led to reduced binding of DNMT1 to the CTCF binding sites at ICR locus, whereas forced expression of FOXA1 in A549 cells facilitated the binding of DNMT1 to these sites (Fig. 4I). Consequently, loss of FOXA1 in PC-9 cells increased CTCF binding to IGF2-ICR, whereas forced expression of FOXA1 in A549 cells reduced CTCF binding to IGF2-ICR (Fig. 4J). Treatment with the DNMT1 inhibitor 5-azacytidine (5-Aza) led to a reduction in *IGF2* mRNA levels in FOXA1-expressing A549, Calu-3, and PC-9 cells (Fig. 4K). Western blot assays revealed similar alterations in IGF2 protein levels upon 5-Aza treatment in LUAD cells (Fig. 4L). We further silenced DNMT1 expression using small interfering RNA (siRNA). As shown in Fig. 4M, N, transient silencing of DNMT1 in FOXA1-expressing A549 and Calu-3 cells repressed IGF2 mRNA and protein expression. Thus, these data indicated that FOXA1 directly bound to the IGF2-ICR and recruited DNMT1 to mediate methylation of CTCF binding sites at IGF2-ICR locus, resulting in LOI of *IGF2*.

**Fig. 5:**
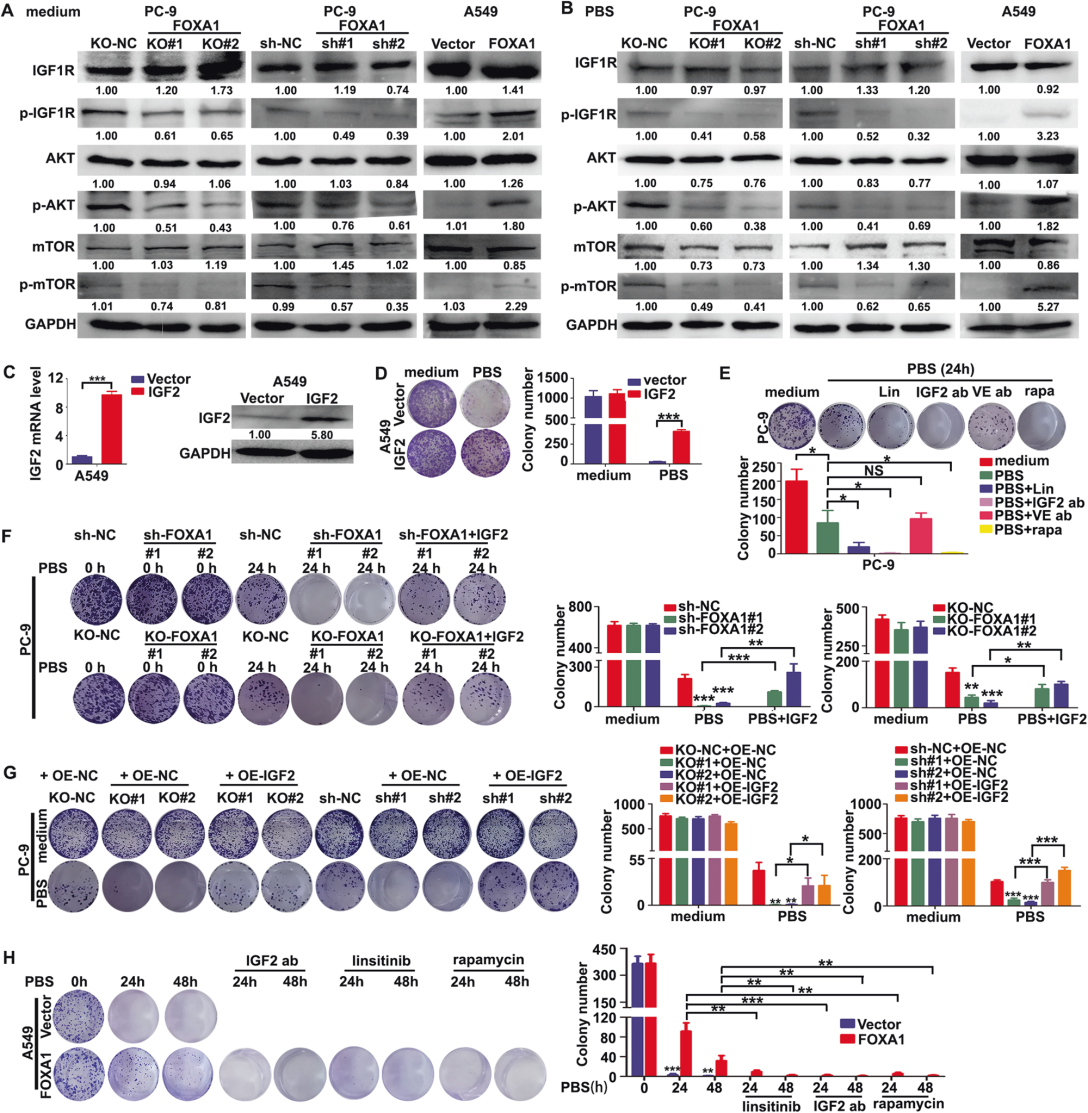
FOXA1 prevented metabolic stress-induced autophagic cell death via activation of IGF2/IGF1R/mTORC1 signaling. **A**, **B** Status of the IGF1R/AKT/mTORC1 signaling pathway upon loss or gain of expression of FOXA in LUAD cells cultured in complete medium or PBS as evaluated by western blot assays. **C** Exogenous IGF2 expression levels in A549 cells as determined by RT-PCR or western blot assays. Mean ± SD, *n* = 3. **D** Survival of A549 cells with or without IGF2 forced expression as measured using colony formation assays. Mean ± SD, *n* = 3. **E** Survival of PC-9 cells in nutrients deprived conditions upon treatment with linsitinib (0.5 μM), anti-IGF2 antibodies (0.5 μg/mL), anti-VEGF antibodies (0.5 μg/mL), or rapamycin (50 nM) were determined using colony formation assays. Mean ± SD, *n* = 3. **F** Colony formation assays demonstrated that supplementation with recombinant IGF2 restored survival in FOXA1-deficient PC-9 cells under metabolic stress conditions. Mean ± SD, *n* = 3. **G** Colony formation assays demonstrated that stable expression of IGF2 restored survival in FOXA1-deficient PC-9 cells under metabolic stress conditions. Mean ± SD, *n* = 3. **H** Colony formation assays indicated that treatment with IGF2 antibody (0.5 μg/mL), VEGF antibody (0.5 μg/mL), linsitinib (0.5 μM) or rapamycin (50 nM) abolished the protective effects of FOXA1 expression on A549 cells in nutrients deprived conditions. Mean ± SD, *n* = 3. *P < 0.05; **P < 0.01; ***P < 0.001, NS not significant.

### FOXA1 prevented nutrients deprivation-induced autophagic cell death through IGF2/IGF1R/mTORC1 signaling

We next examined whether FOXA1 expression affected the downstream signaling of IGF2. As shown in Fig. 5A, CRISPR/Cas9- or shRNA-dependent loss of function of FOXA1 in PC-9 cells led to a reduction in the levels of phosphorylated IGF1R and downstream phosphorylated AKT and mTORC1. By contrast, FOXA1 expression in A549 cells resulted in a significant elevation of phosphorylated IGF1R, AKT, and mTORC1. Neither gain nor loss of function of FOXA1 affected the total protein levels of IGF1R, AKT, and mTORC1. We also measured the status of the IGF2/IGF1R/AKT/mTORC1 signaling pathway when cells were subjected to nutrients deprivation. As shown in Fig. 5B, loss of FOXA1 in PC-9 cells suppressed IGF1R, AKT, and mTORC1 phosphorylation, whereas forced expression of FOXA1 in A549 cells exerted the opposite effects when cells were cultured in PBS. However, forced expression of FOXA1 in Calu-3 cells failed to activate the IGF1R/AKT/mTORC1 signaling pathway under both nutrients-rich and nutrients deprived conditions (Fig. S7A, B).

Next, we examined whether increased IGF2 autocrine confer resistance to autophagic cell death in LUAD cells. As demonstrated by qPCR and western blot assays, overexpression of IGF2 in A549 cells was achieved using an IGF2-expressing lentivirus (Fig. 5C). Forced expression of IGF2 did not affect the growth and colony formation ability of A549 cells under nutrients-rich conditions, but markedly enhanced the survival of A549 cells in PBS (Fig. 5D). By contrast, supplementation with anti-IGF2 antibodies or blocking its downstream signal transduction using linsitinib or rapamycin severely impaired the survival of PC-9 cells in nutrients deprived conditions (Fig. 5E). Supplementation with anti-vascular endothelial growth factor (VEGF) antibodies did not impair PC-9 cell survival under nutrients deprived conditions, suggesting an IGF2-specific role in preventing LUAD cell death in nutrients deprived conditions (Fig. 5E). The exception is that IGF2 overexpression exerted minimal effects on Calu-3 cell growth and survival in both nutrients-rich and nutrients deprived conditions (Fig. S8). Supplementation with recombinant IGF2 in FOXA1-depleted PC-9 cells prevented cell death under nutrients deprivation (Fig. 5F). In addition, re-expression of IGF2 using a lentiviral system in FOXA1-depleted PC-9 cells also rescued cell survival under nutrients deprivation (Fig. 5G). By contrast, supplementation with IGF2-neutralizing antibody or blockage of IGF1R and mTORC1 signaling using linsitinib or rapamycin completely abolished the cytoprotective effects of FOXA1 in A549 cells under nutrients deprivation (Fig. 5H). Thus, these results indicated that FOXA1 expression prevented ACD induced by nutrients deprivation through induction of tumor cell-derived IGF2 and activation of downstream IGF1R/mTORC1 signaling in LUAD cells.

### FOXA1 suppressed autophagic cell death by promoting degradation of lysosomal β-glucocerebrosidase-1 (GBA1) in IGF2 signaling dependent manner

**Fig. 6:**
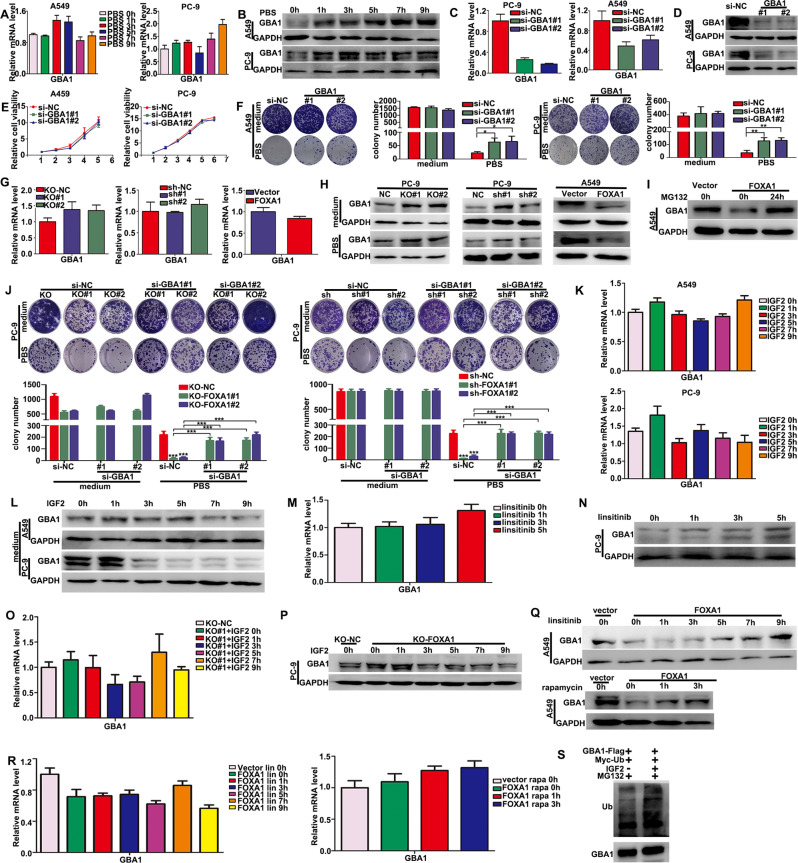
Repression of GBA1 protein by IGF2 signaling contributed to the survival advantage of LUAD cells conferred by FOXA1 under metabolic stress conditions. **A** The mRNA levels of *GBA1* were evaluated by RT-PCR. **B** The protein levels of GBA1 in LUAD cells were measured by western blotting. **C**, **D** The mRNA and protein levels of GBA1 in siRNA-transfected LUAD cells were measured by RT-PCR and western blot assays. Mean ± SD, *n* = 3. **E** Effect of silencing GBA1 on cell growth under nutrient-rich conditions was evaluated by using CCK-8 assays. Mean ± SD, *n* = 5. **F** Effects of silencing GBA1 on survival in LUAD cells under nutrient-rich or starvation conditions were measured using colony formation assays. Mean ± SD, *n* = 3. **G**, **H** The mRNA and protein levels of GBA1 in FOXA1-expressing or FOXA1-deficient cells were measured by RT-PCR and western blot assays. **I** GBA1 protein levels in FOXA1-expressing A549 cells treated with or without MG132 as determined by western blot assays. **J** Colony formation assays. Mean ± SD, *n* = 3. **K**, **L** Effects of IGF2 on GBA1 mRNA and protein levels were determined by RT-PCR or western blot assays. Mean ± SD, *n* = 3. **M**, **N** Effects of IGF2 on GBA1 mRNA and protein levels in FOXA1-depleted PC-9 cells were determined by RT-PCR or western blot assays. **O**, **P** Effects of linsitinib on endogenous GBA1 levels in PC-9 cells were determined by RT-PCR and western blot assays. **Q**, **R** Effects of linsitinib and rapamycin on GBA1 levels in FOXA1-expressing A549 cells were determined by RT-PCR or western blot assays. Mean ± SD, *n* = 3. **S** Ubiquitination of GBA1 was determined by immunoprecipitation and western blot assay. *P < 0.05; **P < 0.01; ***P < 0.001.

**Fig. 7:**
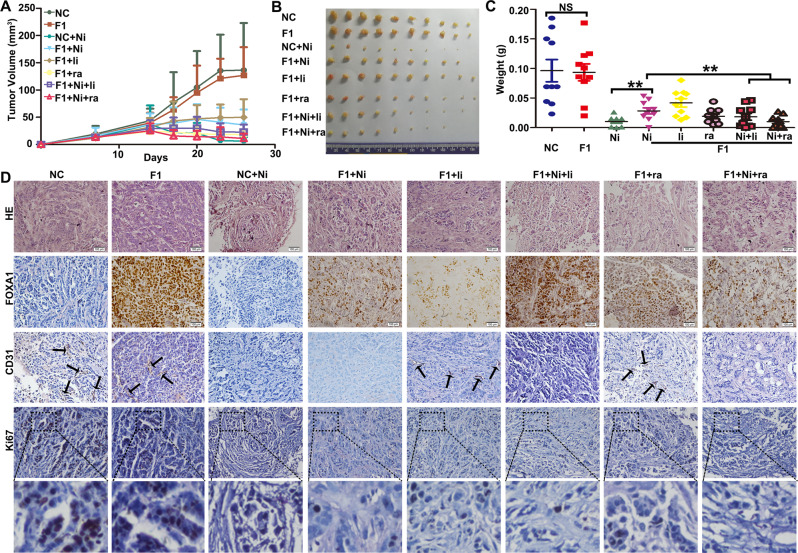
FOXA1 expression reduced the efficacy of nintedanib against xenograft tumors derived from A549 cells. **A** Growth curve of xenograft tumors in nude mice. Mean ± SD, *n* = 10. **B** Macro view of xenograft tumors. **C** Weights of xenograft tumors as measured at the endpoint of the experiment. Mean ± SD, *n* = 10. **D** H&E staining and immunohistochemistry staining of xenograft tumors. Ni nintedanib (100 mg/kg). li linsitinib (25 mg/kg). ra rapamycin (4 mg/kg).

The lysosomal enzyme GBA1 was previously identified as an ACD executor in A549 lung cancer cells [35]. Therefore, we next examined whether GBA1 was involved in nutrients deprivation-induced ACD in lung cancer. We found that nutrients deprivation led to GBA1 protein accumulation in A549 and PC-9 cells, without significantly affecting *GBA1* mRNA levels (Fig. 6A, B). Two specific siRNAs targeting GBA1 successfully decreased GBA1 mRNA and protein levels in A549 and PC-9 cells (Fig. 6C, D). Silencing GBA1 did not affect the growth and survival of A549 and PC-9 cells under nutrients-rich conditions (Fig. 6E, F), but significantly enhanced cell survival in nutrients deprived conditions (Fig. 6F). Neither the inactivation of FOXA1 nor the forced expression of FOXA1 affected *GBA1* mRNA levels in LUAD cells (Fig. 6G). However, the depletion of FOXA1 in PC-9 cells increased GBA1 protein levels in both nutrients enriched and nutrients deprived conditions, whereas forced expression of FOXA1 in A549 cells dramatically reduced GBA1 protein levels (Fig. 6H). Treatment with MG132 restored GBA1 protein levels in FOXA1-expressing A549 cells, suggesting that FOXA1 reduced GBA1 protein levels by enhancing the proteasomal degradation of GBA1 protein (Fig. 6I). Colony formation assays demonstrated that silencing GBA1 in FOXA1-depleted PC-9 cells restored cell survival in nutrients deprived conditions, but had minimal effect on cell growth and survival in nutrients-rich conditions (Fig. 7J), highlighting the crucial role of GBA1 in promoting nutrients deprivation-induced ACD in FOXA1-deficient PC-9 cells.

We then examined how FOXA1 suppressed GBA1 protein levels in LUAD cells. Supplementation with recombinant IGF2 exerted little effect on *GBA1* mRNA levels (Fig. 6K), but decreased GBA1 protein levels in A549 and PC-9 cells (Fig. 6L). By contrast, blocking IGF1R with linsitinib in PC-9 cells did not affect *GBA1* mRNA levels (Fig. 6M), but enhanced GBA1 protein levels (Fig. 6N). Recombinant IGF2 did not significantly change *GBA1* mRNA levels in FOXA1-depleted PC-9 cells (Fig. 6O), but markedly reduced GBA1 protein levels (Fig. 6P). By contrast, linsitinib or rapamycin restored GBA1 protein levels in FOXA1-expressing A549 cells (Fig. 6Q), but did not significantly affect *GBA1* mRNA levels (Fig. 6R). We further demonstrated that IGF2 treatment promoted ubiquitination of Flag-GBA1 protein in HEK293 cells (Fig. 6S). Thus, these results implied that FOXA1 promoted ubiquitin-proteasomal degradation of GBA1 protein in LUAD cells by activating the IGF2/IGF1R/mTORC1 signaling axis.

### FOXA1 expression conferred a survival advantage against anti-angiogenesis therapy in LUAD xenograft tumors

Nutrients deprivation usually occurs in vivo when the blood supply is cut off. The enhanced survival of FOXA1-expressing LUAD cells in nutrients deprived conditions in vitro prompted us to consider that FOXA1 expression may confer a survival advantage against anti-angiogenesis therapy in LUAD cells. Therefore, we employed a xenograft tumor model to study the response of FOXA1-expressing tumors to anti-angiogenesis agents. Forced expression of FOXA1 in A549 cells exerted minimal effect on xenograft tumor growth in nude mice (Fig. 7A). Administration of nintedanib, a tyrosine kinase inhibitor (TKI) that targets the VEGF pathway and is approved for use in the treatment of LUAD [36, 37], effectively suppressed the growth of control tumors. However, FOXA1-expressing tumors were less sensitive to growth inhibition by nintedanib compared with tumors from vector control A549 cells (Fig. 7A). Combined treatment of nintedanib with either IGF1R inhibitor linsitinib or mTORC1 inhibitor rapamycin suppressed the growth of FOXA1-expressing tumors with more potency than treatment with nintedanib alone (Fig. 7A–C). Hematoxylin and eosin (H&E) staining indicated that nintedanib treatment severely disrupted the architecture of control tumors, but had weaker effects on FOXA1-expressing tumors. Furthermore, combined treatment of nintedanib with linsitinib or rapamycin exerted stronger disruptive effects on FOXA1-expressing tumors (Fig. 7D). Immunohistochemistry assays revealed that administration of nintedanib potently reduced the density of microvessels marked by CD31 in xenograft tumors (Fig. 7D). As expected, combining nintedanib with either linsitinib or rapamycin significantly reduced Ki-67^+^ cells in FOXA1-expressing tumor sections (Fig. 7D).

### Overexpression of FOXA1 was associated with poor prognosis in patients with LUAD receiving anti-angiogenesis therapy

**Fig. 8:**
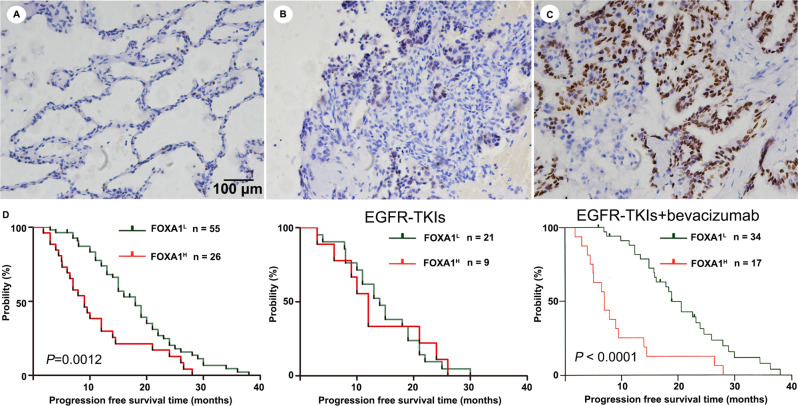
High levels of FOXA1 protein corresponded to unfavorable progression-free survival in patients with LUAD who underwent treatment with a combination of EGFR-TKI and bevacizumab. **A** Negative staining of FOXA1 protein in lung tissues. **B** Negative or weak staining of FOXA1 protein in LUAD samples. **C** Intense nuclear staining of FOXA1 protein in LUAD samples. **D** Progression-free survival in patients with LUAD patients receiving EGFR-TKI treatment alone or EGFR-TKI combined with bevacizumab stratified according to FOXA1 protein. Scale bar: 100 μm.

Since FOXA1 reduced the efficacy of anti-angiogenesis therapy against experimental lung xenograft tumor, we assumed that it expression level might be related to the efficacy of anti-angiogenesis therapy in LUAD patients. We measured FOXA1 protein levels in LUAD patients of advanced stage who received anti-angiogenesis therapy combined with EGFR-TKI or EGFR-TKI treatment alone. As shown in Fig. 8A, FOXA1 protein is not expressed in normal lung tissues and 55 of 81 (67.9%) tumors from LUAD patients of advanced stage (Fig. 8A, B), whereas is highly expressed in 26 of 81 (32.1%) tumors from LUAD patients of advanced stage (Fig. 8C). High expression of FOXA1 protein was associated with shorter relapse-free survival in patients of advanced stage (Fig. 8D). Among these 81 LUAD patients of advanced stage, 51 patients received bevacizumab treatment combined with EGFR-TKIs, whereas 30 patients received EGFR-TKIs treatment alone. FOXA1 expression was significantly associated with shorter relapse-free survival in patients of advanced stage who received bevacizumab treatment combined with EGFR-TKIs, but not associated with relapse-free survival in LUAD patients of advanced stage who received EGFR-TKI treatment alone (Fig. 8D), implying that overexpression of FOXA1 probably contributes to tumor cells survival during anti-angiogenesis therapy.

## Discussion

In this study, we uncovered a previously unrecognized role of FOXA1 in protecting LUAD cells from nutrients deprivation-induced ACD, a process in which the IGF2/IGF1R/mTORC1 signaling cascade and ubiquitin-proteasome-dependent degradation of GBA1 are involved (Fig. S9). Inhibition of ACD induced by nutrients deprivation probably confer FOXA1^high^ expressing LUAD cells survival advantage during anti-angiogenesis, whereas it could be reversed by inhibition of IGF2/IGF1R/mTORC1 signaling cascade.

The proliferation of transformed cells is determined by nutrients availability. Inadequate tumor perfusion results in that nutrients are often in short supply, which is an obstacle that the transformed cells have to face [38]. Tumor cells initiate autophagy in response to metabolic stresses such as nutrient deprivation [39]. Autophagy induced by metabolic and therapeutic stresses may exert a prosurvival or prodeath role [40, 41]. The effect of stresses-induced autophagy on cell survival is related to the extent and duration of stresses. Autophagy may ensure cell growth and survival in response to transient stresses through self-digestion of unnecessary proteins and damaged organelles. However, if stresses persist for longer, persistent or excessive autophagy may cause cell death. In certain circumstances, induction of autophagic cell death is the major mechanism that some anticancer agents exert the therapeutic effects against cancer [42]. Oncogenic proteins may promote tumor cell survival in starvation via inhibition of autophagic cell death [43, 44]. In this study, we reported that the expression of FOXA1 promotes LUAD survival in nutrients deprived conditions through inhibiting autophagic cell death, which is in accordance with the oncogenic role of FOXA1 in LUAD development [22, 23]. A pilot study revealed that the expression level of FOXA1 is elevated in subpopulations of the A549 lung cancer cell line with high invasive potential. Silencing FOXA1 attenuates invasiveness and proliferation of the highly invasive A549 subpopulations even in conditions without serum starvation [45], suggesting that FOXA1-expressing lung cancer cells have survival advantage or proliferative phenotypes without treatment of serum starvation [45]. They did not investigate whether silencing FOXA1 affect autophagy machinery of the highly invasive A549 subpopulations. In this study, we measured the effect of FOXA1 expression on lung cancer cells survivability upon long term PBS treatment, which was routinely used to induce autophagy. We demonstrated that FOXA1 enhanced lung cancer cell survival upon nutrients deprived conditions through suppressing autophagic cell death. Thus, our data along with the previous finding indicate that FOXA1 is a bona fide oncogenic factor contributing to lung cancer cell survival both in nutrients enriched and nutrients poor conditions. It is worth noting that serum starvation may result in de-phosphorylation of many numbers of molecules inside the cells. We do observe that FOXA1 expression help to maintain the phosphorylation status of IGF1R signaling cascade of lung cancer cells in the serum starvation conditions, which might be explained by increased IGF2 autocrine in lung cancer cells by FOXA1.

Another study reported that high expression of FOXA1 contributes to acquisition of chemo-resistance in LUAD cells through inhibiting apoptosis induced by anticancer drug docetaxel [46]. It has been shown that autophagy may be involved to prevent apoptosis upon docetaxel treatment [47]. However, autophagy may also promote apoptosis in some circumstances [48, 49]. In this study, we did not investigate whether FOXA1 affect LUAD cells sensitivity to docetaxel. So, it is difficult to speculate whether autophagy regulated by FOXA1 is involved in the process of acquisition of docetaxel-resistance in LUAD cells. On the other hand, cancer cells may initiate various cell death pathways in response to different death stimuli. Induction of autophagic cell death may be the major mechanism leading to cell death in some apoptosis-resistant cancers [50]. We considered that FOXA1 may prevent different forms of cell death induced by different death stimuli by activating various target genes in LUAD cells. Though the essence of FOXA1 expression for the lung cancer cells survival has been reported in the previous publications, our work highlighted an unusual role of FOXA1 in suppressing autophagic cell death of LUAD cells in nutrients poor conditions. A recent study found that FOXA1 overexpression suppresses interferon signaling and promotes cancer immuno- and chemotherapy resistance in prostate cancer and bladder cancer [51]. Whether FOXA1 overexpression promotes immune evasion in lung adenocarcinoma needs to be further explored.

We provide evidence that the protective role of FOXA1 on LUAD cells is highly dependent on induction of IGF2, which is a multifunctional growth factor that promotes tumor cell survival via activating IGF1R signaling cascade [52]. We also demonstrated that blockage of IGF1R or its downstream mTORC1 signaling diminished the protective effect of FOXA1 on LUAD cells in nutrient-depleted conditions in vitro, suggesting that targeted inhibition of IGF1R or mTORC1 might be a precision therapy strategy for FOXA1-driven LUAD. Although FOXA1 induced IGF2 expression in Calu-3 cells, it failed to increase Calu-3 cell survival in response to nutrients deprivation. Indeed, forced expression of IGF2 also failed to increase Calu-3 cell survival in nutrients deprived conditions. This may be due to Calu-3 cells have high levels of *IGF2R* mRNA (Fig. S10), which lacks tyrosine kinase activity, and its binding with IGF2 would reduce the bioavailability of IGF2 [53].

The *IGF2* is the classic imprinted gene. The imprinting of *IGF2* is maintained by binding of the insulator protein CTCF to unmethylated maternal chromosome, which remodels chromatin structure to form insulator elements, thus preventing enhancers downstream from *H19* to activate *IGF2* on the maternal chromosome [54]. Overexpression of IGF2 is frequent in human cancers and is associated with a poor prognosis [55]. The LOI of the *IGF2* gene is the most common epigenetic alteration leading to aberrant activation of normally silent maternally inherited alleles in LUAD [12, 13]. LOI of *IGF2* in lung cancer is mediated by DNMT1 [34]. There is minimal data on the mechanism of initiation of the LOI of *IGF2* in lung cancers. We identified that there is a FOXA1 binding site adjacent to the seven CTCF binding sites at IGF2-ICR locus. We demonstrated that FOXA1 interacted with DNMT1 and recruited DNMT1 to catalyze methylation of CpG-rich regions at IGF2-ICR, which in turn results in disruption of CTCF binding at IGF2-ICR and leads to activation of transcription of *IGF2*. Our findings provided the first evidence that FOXA1 initiates DNMT1-mediated LOI of *IGF2* in in human LUAD through directly binding to IGF2-ICR locus.

GBA1 is a lysosomal hydrolase that catalyzes the conversion of glucosylceramide to glucose and ceramide [56]. GBA1 is required for lysosomal function and autophagolysosome formation [57]. It has been demonstrated previously that GBA1 acts an ACD executor in A549 lung cancer cells [35]. In this study, we showed that IGF2 accelerated the ubiquitination of the GBA1 protein. FOXA1 promoted the degradation of GBA1 protein in LUAD cells by activating the IGF2/IGF1R/mTORC1 signaling pathway. Silencing GBA1 in FOXA1-deficient PC-9 cells attenuated ACD in nutrients deprived conditions, indicating GBA1 is responsible for execution of ACD in this context. To date, the E3 ligase TRIP12 has been identified as the major E3 ligase governing GBA1 stability [58]. The mTORC1 signaling is a central regulator of autophagy by modulating multiple aspects of the autophagy process. It has been shown that phosphorylation of RNF168 at Ser60 by mTORC1-S6K pathway promotes TRIP12-mediated RNF168 degradation [58]. We assumed that GBA1 might be a substrate of mTORC1. Phosphorylation of GBA1 by mTORC1 probably facilitates subsequent ubiquitination of GBA1 mediated by TRIP12. However, this assumption needs to be elucidated.

The tumor microenvironment is a highly unfavorable metabolic milieu characterized by poor nutrient availability [59]. Autophagic cell death may occur in vivo when blood supply was totally blocked. For example, neurons undergo autophagic cell death in cerebral ischemia [60]. Nutrient deprivation is an inevitable consequence that tumor cells have to face when the blood supply was cut off by angiogenesis inhibitors. Because FOXA1 enhanced LUAD cells survival in nutrients deprived conditions in vitro, we hypothesized that FOXA1 might influence the xenograft tumor survival in vivo when the nutrients supply in tumors was cut off by anti-angiogenic therapy. As expected, we found that FOXA1^high^ xenograft tumors were less sensitive to anti-angiogenesis reagent nintedanib in vivo. The expression level of FOXA1 protein associates with unfavorable prognosis in LUAD patients of advanced stage who received combinatory treatment of bevacizumab along with EGFR-TKIs, but not with those who received EGFR-TKIs alone, suggesting FOXA1-driven LUAD probably are refractory to anti-angiogenesis therapy. Combinatory treatment using the IGF1R inhibitor linsitinib or the mTORC1 inhibitor rapamycin with nintedanib more efficiently eradicated FOXA1^high^ xenograft tumors than nintedanib alone. Our study is in consistent with others observation that IGF1R inhibitor potentiates the efficacy of Anti-VEGF therapy against IGF2-Overexpressing colorectal cancer tumors [61]. It has been shown that angiogenic environment induced by VEGF treatment inhibits autophagic cell death [62]. Our study also implies that enhancing autophagic cell death by inhibiting IGF2/IGF1R/mTORC1 signaling might be an alternative strategy to improve the efficacy of anti-angiogenesis therapy against FOXA1-driven LUAD.

One limitation of this study is that in vitro nutrients deprivation could not mimic hypoxia conditions induced by anti-angiogenesis therapy in vivo. It is not clear whether FOXA1 would affect LUAD cells response to hypoxia. Furthermore, stromal cells in tumor environments may interact with tumor cells to regulate cellular responses to stress stimuli. Patient-derived organoids containing stromal cells may be an alternative strategy to study the effects of FOXA1 expression on LUAD cell survival under starvation conditions.

## Conclusions

In this study, we uncovered a previously unrecognized role of FOXA1 expression in protecting LUAD cells from starvation-induced autophagic cell death by mediating the LOI of *IGF2*. FOXA1 expression conferred tolerance to anti-angiogenesis therapy in LUAD tumors in vivo, and this effect could be reversed by blocking IGF2 signaling. Our study may help facilitate the development of precision targeted therapies for patients with LUAD.

## Supplementary information

Supplemental data

full length uncropped original western blots

Reproducibility checklist

## Data Availability

All data generated or analyzed during this study are included in this published article.

## Material availability

The materials used and/or analyzed during the current study are available from the corresponding author on reasonable request.
