# Supplementary material for: FOXA1 prevents nutrients deprivation induced autophagic cell death through inducing loss of imprinting of IGF2 in lung adenocarcinoma
Source: Cell Death Dis. 2022 Aug 16;13(8):711. doi: 10.1038/s41419-022-05150-8 (PMC9381574; doi:10.1038/s41419-022-05150-8)
Supplement: Supplementary file 1 — Supplemental data [file 41419_2022_5150_MOESM1_ESM.docx]

**Supplementary Figure legends**


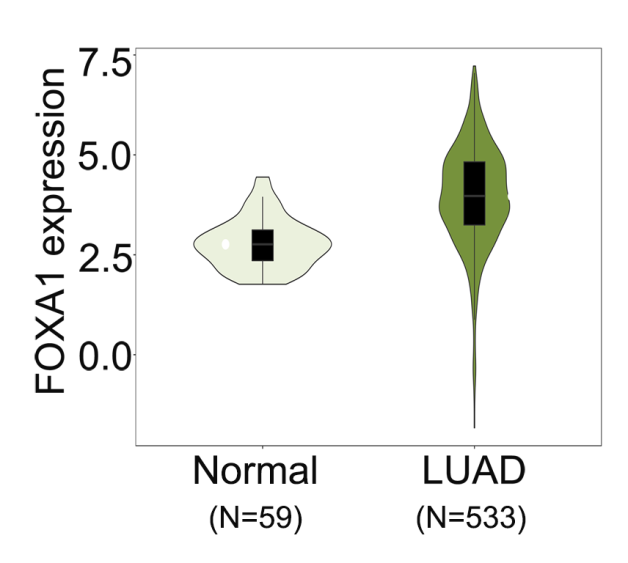


**Figure S1 FOXA1 mRNA was increased in LUAD samples.**

The levels of FOXA1 mRNA in LUAD samples were analyzed by RNA-seq. Data were collected from TCGA.


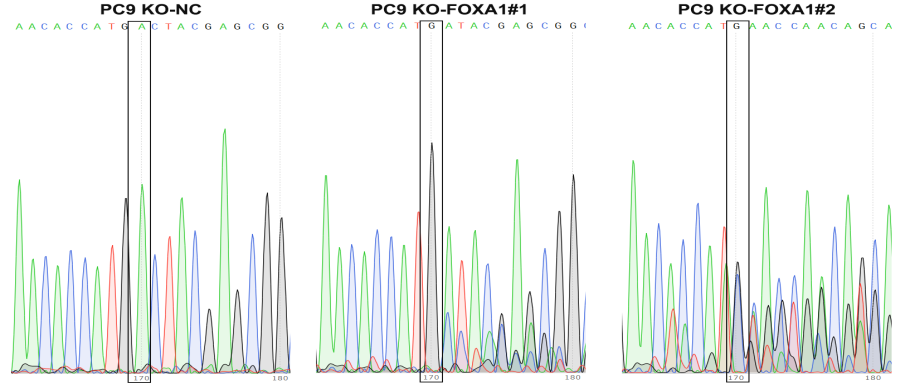


**Figure S2 Genome sequencing analysis of FOXA1 knockout in PC-9 cells by CRISPR/Cas9.**

Genomic DNA were extracted and the fragment flanking the targeting sequence were amplified by PCR. Sanger sequencing revealed gene editing by CRISPR/Cas9 introduce heterozygous frame-shift mutation in *FOXA1*.

**
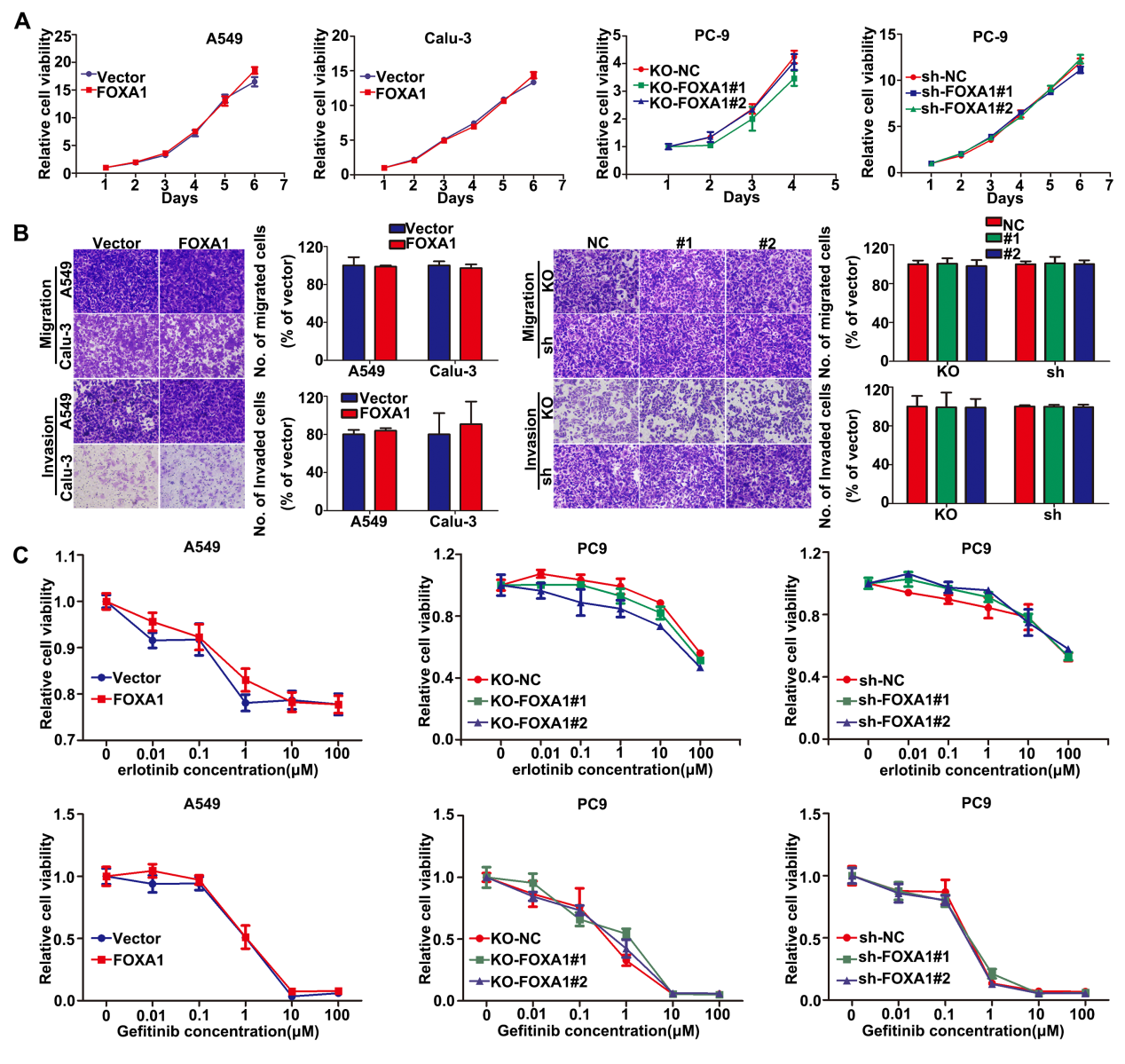
**

**Figure S3 Expression of FOXA1 in LUAD cells exerted little effects on cell growth, invasiveness and sensitivity against EGFR-TKI.**

A, cell growth of LUAD cells were determined by CCK-8 assays. Mean±SD, *n*=5. B, migration and invasiveness of LUAD cells were evaluated by transwell assays. Mean±SD, *n*=3. C, cell responses to EGFR-TKI were measured by CCK-8 assays. Mean±SD, *n*=5.

**
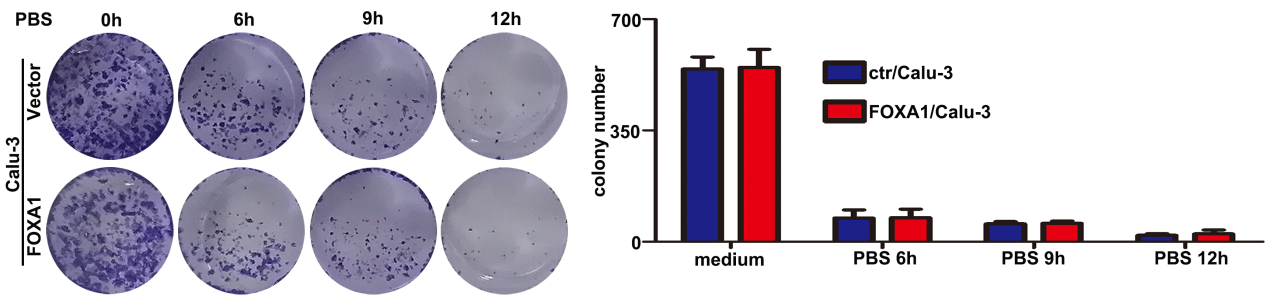
**

**Figure S4 Expression of FOXA1 in Calu-3 cell did not affect cell survival in starvation.**

Cell survival of FOXA1-expressing Calu-3 cells was determined by colony formation assays. Mean±SD, *n*=3.

**
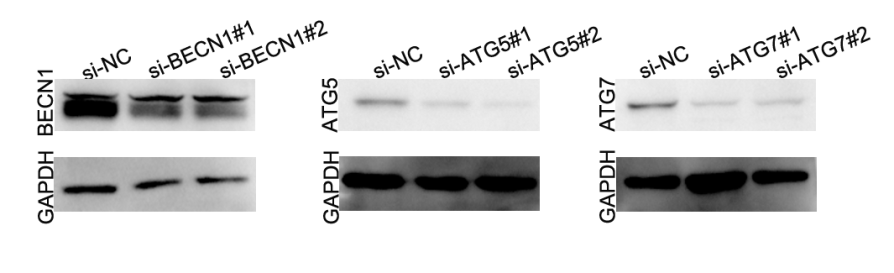
**

**Figure S5 Silencing ATGs in LUAD cells.**

The protein levels of ATGs in siRNAs transfected cells were determined by western blot assays.


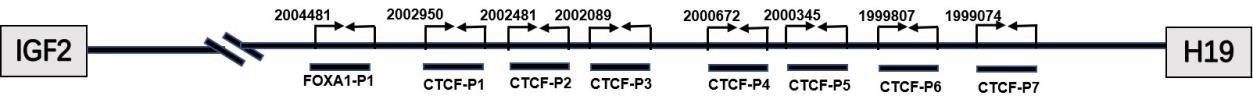


**Figure S6 Location of primer sets used in ChIP-PCR assays at IGF2-ICR locus.**

Different primer sets were used to amplify the fragments containing a FOXA1 binding site or seven CTCF binding sites at IGF2-ICR. The location of fragments amplified by different primer sets were showed in the schematic diagram.

**
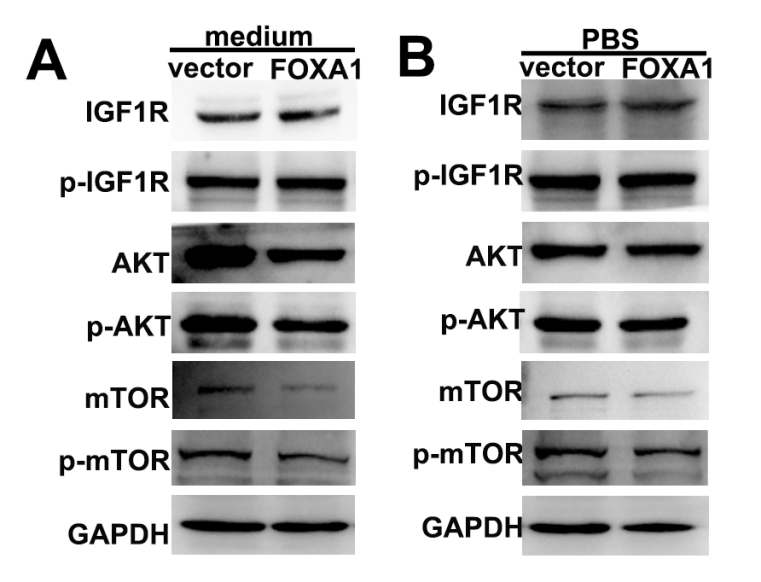
**

**Figure S7 Forced expression of FOXA1 in Calu-3 cell exerted little effects on IGF1R/mTORC1 signaling.**

A, The levels phosphorylated or of total proteins of IGF1R, Akt and mTORC1 in FOXA1-expressing Calu-3 cell cultured in completed medium or PBS solutions were determined by western blot assays.

**
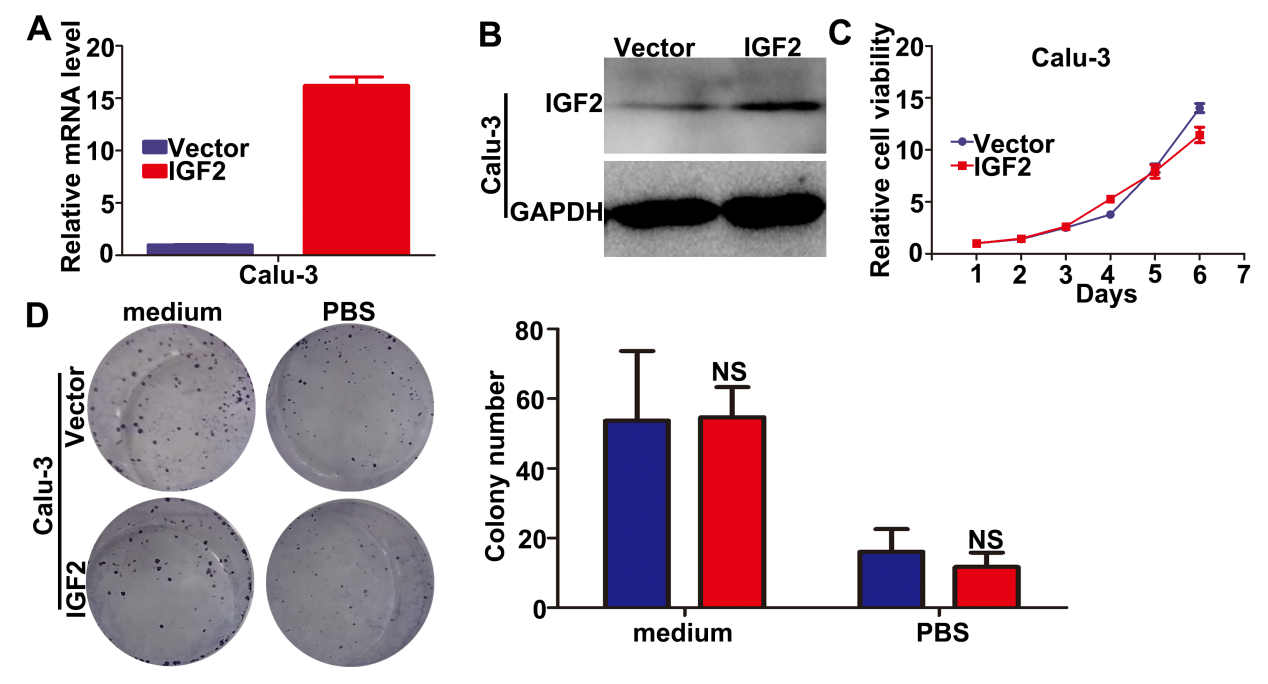
**

**Figure S8 Forced expression of IGF2 in Calu-3 cell failed to protect cell from metabolic stress.**

A&B, the levels of IGF2 mRNA or protein in Calu-3 cells were measured by RT-PCR or western blot assays. C, cell growth was evaluated by CCK-8 assays. Mean±SD, *n*=5. D, cell survival in complete medium or starvation conditions were determined by colony formation assays. Mean±SD, *n*=3. NS, non significant.


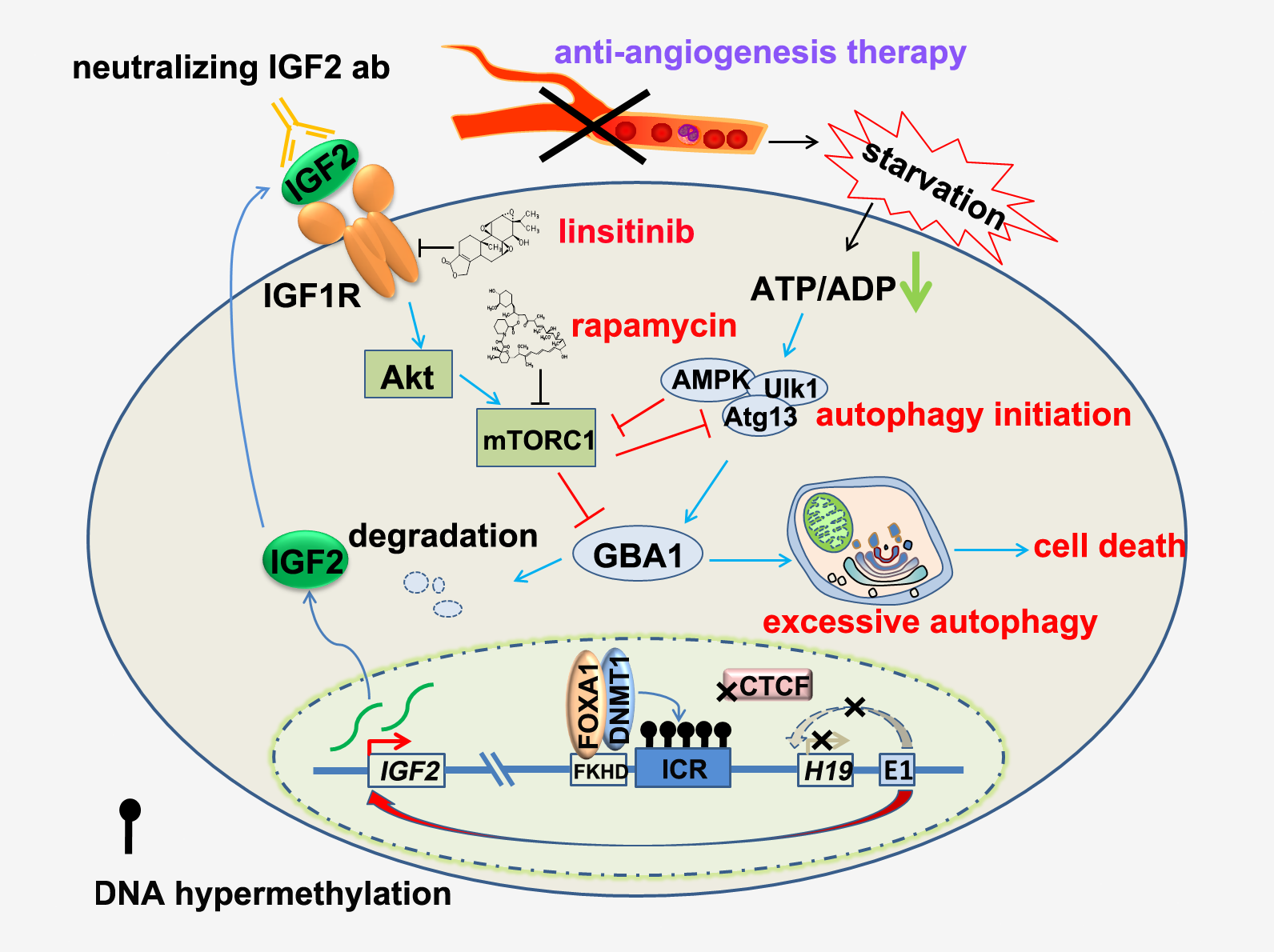


**Figure S9. Schematic diagram illustrating the roles of FOXA1 in starvation-induced autophagic cell death and anti-angiogenesis therapy in LUAD.**

FOXA1 interacts with DNMT1 and recruits DNMT1 to the ICR of the *IGF2* gene locus, resulting in loss of imprinting of *IGF2* and upregulation of *IGF2* in LUAD cells. Autocrine of IGF2 activates IGF1R/AKT/mTORC1 signaling and inhibits basal of autophagy in LUAD cells. GBA1 is a mediator of autophagic cell death induced by metabolic stress. Activation of IGF1R/AKT/mTORC1 signaling leads to destabilization of GBA1 protein, thereby inhibiting metabolic stress-induced autophagic cell death in LUAD cells. Blockade of IGF2/IGF1R/mTORC1 signaling abolishes the cell survival advantage conferred by FOXA1 in LUAD under metabolic stress conditions. High expression of FOXA1 protein hampers the efficacy of anti-angiogenesis therapy against LUAD.


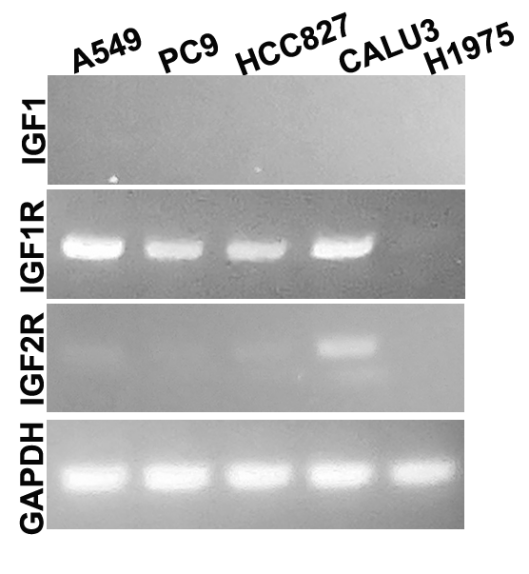


**Figure S10 Expression levels of IGF2 receptors in LUAD cell lines.**

The levels of IGF1, IGF1R and IGF2R mRNA levels in LUAD cells were measured by RT-PCR.

**Table S1** **The sequences of sgRNAs, shRNAs or siRNAs used in this study**

| name | sequence |
| --- | --- |
| FOXA1-sgRNA-F | CACCGCATGTTGCCGCTCGTAGTCA |
| FOXA1-sgRNA-R | AAACTGACTACGAGCGGCAACATGC |

| Scrambled siRNAs | UUCUCCGAACGUGUCACGUTT |
| --- | --- |
| FOXA1#1 siRNA | GAGAGAAAAAAUCAACAGC |
| GBA1#1 siRNA | AGCAGCTAAATATGTTCATGGCATT |
| GBA1#2 siRNA | GCTAAATATGTTCATGGCATTGCTG |
| BECN1#1 siRNA | CCACUCUGUGAGGAAUGCACAGAUA |
| BECN1#2 siRNA | GGAUGAUGAGCUGAAGAGUGUUGAA |
| ATG5#1 siRNA | CCTTTGGCCTAAGAAGAAA |
| ATG5#2 siRNA | CATCTGAGCTACCCGGATA |
| ATG7#1 siRNA | GGAGTCACAGCTCTTCCTT |
| ATG7#2 siRNA | CAGCTATTGGAACACTGT |
| DNMT1#1 siRNA | GGAACCAAGCAAGAAGTGA |
| DNMT1#2 siRNA | AUUACGUAAAGAAGAAUUATT |

**Table S2**  **The primers information used in this study**

| Primers | Sequence (5’-3’) |
| --- | --- |
| ***ChIP-qPCR primers*** |  |

| CTCF-P1-F | | GGAGACCTGGGACGTTTCTG |
| --- | --- | --- |
| CTCF-P1-R | | AGAATCGGCTGTACGTGTGG |
| CTCF-P2-F | | AATGTGGCTCCCATGAGTGT |
| CTCF-P2-R | | TGGTGCAGAATCGGTTGTAGT |
| CTCF-P3-F | | CCATGAGCGTCCTATTCCCA |
| CTCF-P3-R | | TGTGGACTCAAAAGTGGCCG |
| CTCF-P4-F | | CCCTGATGACCCCCGTGAAC |
| CTCF-P4-R | | GGTTGTAGCTGTGGAATCGGA |
| CTCF-P5-F | | GGGCTCAGGCTGTGATGTG |
| CTCF-P5-R | | CCCGGATGGCACAGAATTGG |
| CTCF-P6-F | | TGTGGATAATGCCCGACCTGA |
| CTCF-P6-R | | ACGGAATTGGTTGTAGTTGTG |
| CTCF-P7-F | | CACAGAATCGGTTGTGGCTG |
| CTCF-P7-R | | TGGACCCCCAAGTTAGGAGA |
| IGF2-ICR-FOXA1BS-F | | CCTGTTGGGCGGTTAGAC |
| IGF2-ICR-FOXA1BS-R | | GGAAGCAGAAACTGGGCA |
| NC-F | | CCACCCTCCACCCTTCAA |
| NC-R | | CCAAACTAAAGCAGGAAC |
| ***RT-PCR primers*** | |  |
| FOXA1-qPCR-F | TCCAGGATGTTAGGAACTGTGA | |
| FOXA1-qPCR-R | CCGCTCGTAGTCATGGTGTT | |
| GAPDH-qPCR-F | aacggatttggtcgtattgg | |
| GAPDH-qPCR-R | ttgattttggagggatctcg | |
| GBA1-qPCR-F | GGCAGCCTCACAGGATTG | |
| GBA1-qPCR-R | CCTCCAAATCCCTTCACTT | |
| IGF2-qPCR-F | GTGGGCAAGTTCTTCCAATAT | |
| IGF2-qPCR-R | GGGTGGGTAGAGCAATCAGG | |
| DNMT1-qPCR-F | TATCCGAGGAGGGCTACCTG | |
| DNMT1-qPCR-R | CTCCATCGGACTTGCTCCTC | |
| IGF1R-qPCR-F | GAGGGTTGGTGATTATGCT | |
| IGF1R-qPCR-R | ACTTATTGGCGTTGAGGTAT | |
| IGF2R-qPCR-F | AGGATAACTCCACCTACAACT | |
| IGF2R-qPCR-R | TCTGTATCCGTCGTTGTCT | |

**Table S3 antibodies used in this study**

| name | company | Cat No. |
| --- | --- | --- |
| FOXA1 | Abcam | Ab170933 |
| mTOR | abclonal | A2445 |
| p-mTOR | abclonal | A90978 |
| AKT | CST | 2920S |
| p-AKT | abclonal | A90140 |
| IGF1R | CST | 9750T |
| p-IGF1R | CST | 3024T |
| CD31 | abcam | Ab281583 |
| GBA1 | abclonal | A19057 |
| Ki67 | ZSGB-BIO | ZA-0502 |
| DNMT1 | Active motif | 39204 |
| GAPDH | abclonal | AC001 |
| CTCF | Active motif | 61311 |
| H3K4me1 | abclonal | A2355 |
| H3K27ac | abclonal | A7253 |
| ATG5 | CST | 12994S |
| ATG7 | CST | 8558S |
| BECN1 | abclonal | A7353 |
| LC3B | abclonal | A19665 |
| Ub | Santa cruz |  |
| IGF2 | Abcam | Ab9574 |
| ProteinFind® Goat Anti-Rabbit IgG (H+L), HRP Conjugate | TRAN | HS101-01 |
| ProteinFind® Goat Anti-Mouse IgG (H+L), HRP Conjugate | TRAN | HS201-01 |

**Table S4** **Target genes regulated by FOXA1 in LUAD cells**

| genes upregulated by FOXA1 | TSPAN7; RNF152; NOS1; PPP4R4; SERPINA1; SERPINA10; PAX5; IL31RA; F5; NRN1; AGR2; TFF1; SDR16C5; CPA6; CCR7; VSIG1; PID1; CYTH4; BCAS1; RND1; IGF2; MUC5AC; KPNA7; ACKR3; SIDT1; S100P; CAPN8; KCNK6; CGNL1; PRSS3; LIPG; |
| --- | --- |
| genes downregulated by FOXA1 | 4-Mar; TLE4 |
