# Supplementary figures and images for: FOXA1 prevents nutrients deprivation induced autophagic cell death through inducing loss of imprinting of IGF2 in lung adenocarcinoma
Source: Cell Death Dis. 2022 Aug 16;13(8):711. doi: 10.1038/s41419-022-05150-8 (PMC9381574; doi:10.1038/s41419-022-05150-8)

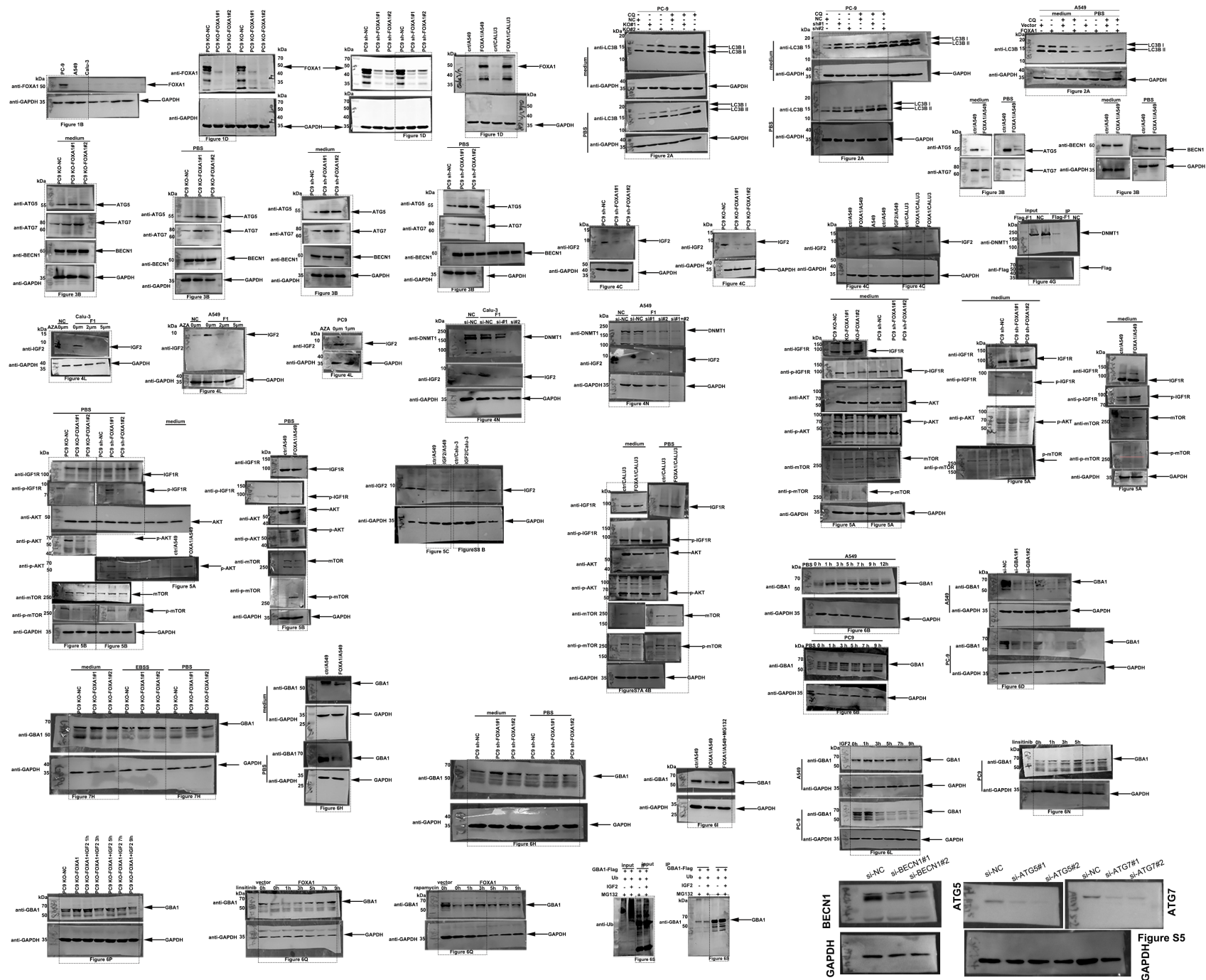

Supplement: Supplementary file 2 — full length uncropped original western blots [file 41419_2022_5150_MOESM2_ESM.pdf]
